# Profiling of the Klebsiella pneumoniae Phosphoproteome under Iron-Limited and Iron-Replete Conditions

**DOI:** 10.1128/mra.00186-23

**Published:** 2023-06-26

**Authors:** Chelsea Reitzel, Arjun Sukumaran, Benjamin Muselius, Siobhan O’Connor, Jennifer Geddes-McAlister

**Affiliations:** a Department of Molecular and Cellular Biology, University of Guelph, Guelph, Ontario, Canada; University of Notre Dame

## Abstract

Klebsiella pneumoniae was compared across iron-limited and iron-replete conditions to assess changes within the phosphoproteome using quantitative mass spectrometry. These comparative proteomic data provide insights into cellular responses to nutrient limitation and how nutrient requirements may be exploited to provide potential antimicrobial targets.

## ANNOUNCEMENT

Klebsiella pneumoniae is found ubiquitously across the environment and within hospital settings (1, 2). The bacterium typically colonizes the gastrointestinal tract or skin of healthy individuals without resulting in infection (1, 3). However, K. pneumoniae is an opportunistic pathogen that is capable of infecting immunocompromised individuals or causing coinfections (1). Importantly, the rise of multidrug resistance and the designation of carbapenemase-producing K. pneumoniae as a high-priority antibiotic-resistant bacterium (4) support the urgent need to find new strategies to neutralize this pathogen.

Iron is the most abundant transition metal for bacteria (5); it is incorporated into proteins to support physiological processes (6), including cellular respiration, nucleic acid synthesis, and metabolism (6–9). Previous proteomic and secretomic profiling of K. pneumoniae under iron-limited conditions identified changes in biological processes associated with iron acquisition and transport, as well as siderophore production (10). To date, the phosphoproteome of K. pneumoniae under these conditions has yet to be explored but presents an opportunity to provide insights into the regulation of cellular signaling events in response to hostile conditions imitating those of host colonization.

**FIG 1:**
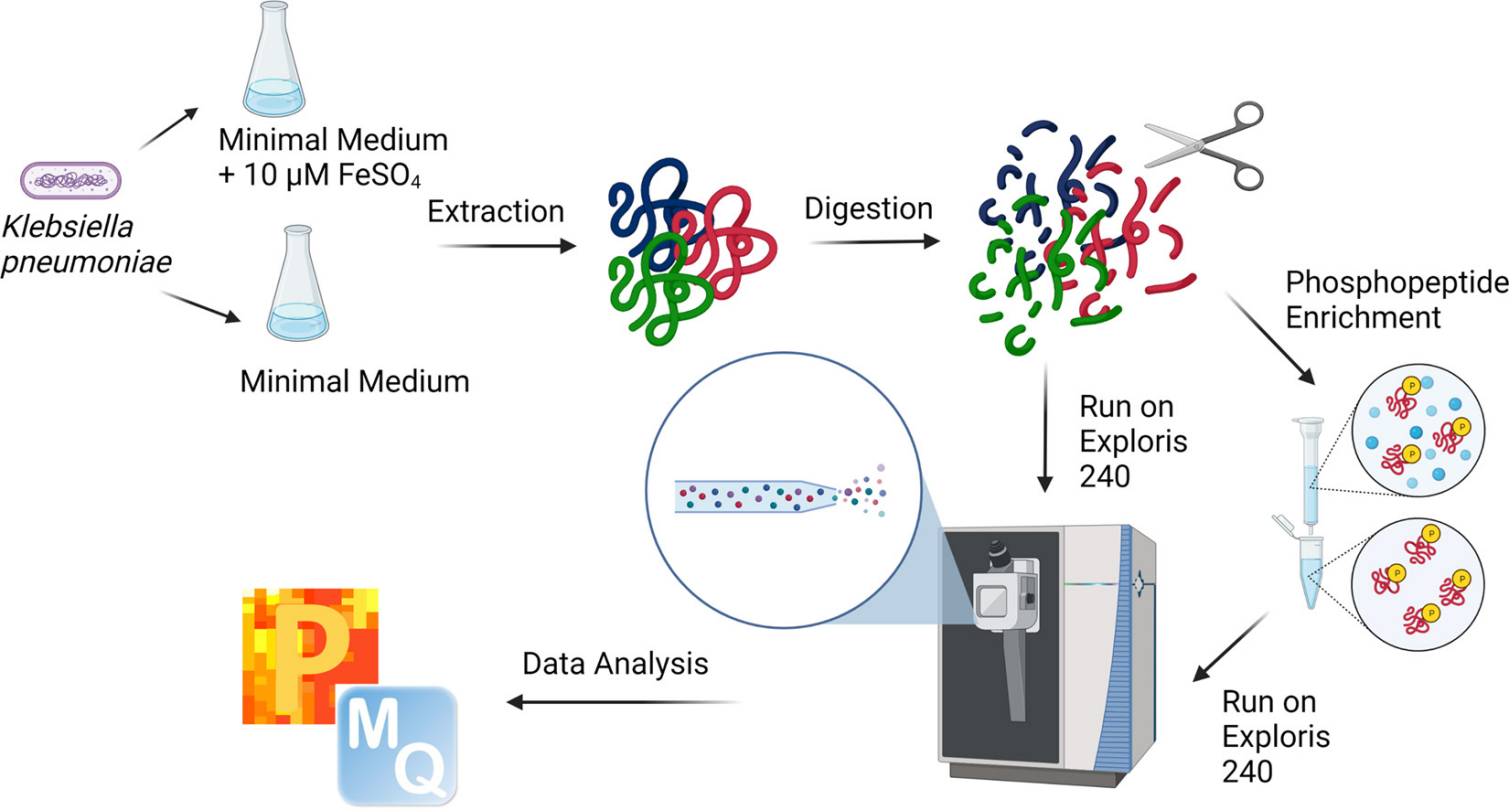
Workflow for bottom-up proteomic profiling of the K. pneumoniae phosphoproteome under iron-limited versus iron-replete conditions. The total proteome was measured for abundance normalization. The figure was generated with BioRender.

Profiling of the total proteome and phosphoproteome of laboratory adapted K. pneumoniae (ATCC 700721) was performed under iron-limited and iron-replete conditions (Fig. 1). K. pneumoniae was grown overnight at 37°C in 5 mL Luria-Bertani (LB) medium, with shaking (200 rpm) (in quadruplicate); 0.5 mL of culture was collected by centrifugation at 3,500 × *g* and washed twice with 0.5 mL M9 minimal medium (6.78 g/L Na_2_HPO_4_, 3 g/L KH_2_PO_4_, 0.5 g/L NaCl, 1 g/L NH_4_Cl, 0.4% [wt/vol] glucose, 2 mM MgSO_4_, and 0.1 mM CaCl_2_, made to 1 L using Chelex 100-treated distilled H_2_O as described previously [11]) to remove any trace of LB broth. Washed cells were subcultured (1:100) at 37°C in 50 mL M9 minimal medium or medium supplemented with 10 μm iron (FeSO_4_), with shaking (200 rpm). At mid-log to early stationary phase (approximately 9 h) (10), cells were collected, pelleted at 1,300 × *g*, and washed twice with 5 mL phosphate-buffered saline (PBS) (10).

For proteomic analyses, cell pellets were processed as described previously (10). Briefly, cells were resuspended in 100 mM Tris-HCl (pH 8.5) with 2% sodium dodecyl sulfate (SDS) (final concentration) and proteinase inhibitor and phosSTOP tablets. Cells were lysed using probe sonication (30 s on/30 s off in an ice bath, with 30% power), reduced with 10 mM dithiothreitol for 10 min at 95°C (800 rpm), alkylated using 55 mM iodoacetamide for 20 min at room temperature in the dark, and precipitated overnight in acetone (final concentration, 80%) at −20°C. Precipitated proteins were pelleted by centrifugation at 13,500 rpm for 10 min and resuspended in 8 M urea-40 mM HEPES buffer, and the protein concentration was assessed (12). Samples were digested overnight at room temperature with trypsin and LysC, at a trypsin-LysC/protein ratio of 1:50. A 10% aliquot of sample (~80 μg) was collected and stored on ice; the remaining sample was subjected to phosphopeptide enrichment (catalog number A32993; Thermo Fisher Scientific) using TiO_2_ columns according to the manufacturer’s instructions. Total peptides and phospho-enriched peptides were purified on STop And Go Extraction (STAGE) tips (13), and 3 μg of peptides were loaded onto Evotips (14) according to the manufacturer’s instructions and separated by Evosep One liquid chromatography system coupled with Thermo Scientific Orbitrap Exploris 240. Mass spectrometry analysis was performed in HCD mode using a 44-min gradient (88 min for the total proteome) with a 15-cm PepSep column with 150 μm diameter and 1.9 μm beads (Evosep, EV1106). The precursor range was set at *m*/*z* 400 to 2,000, with a resolution of 60,000 and an intensity threshold of 2.5E4. Dynamic exclusion was set to 10 sec and charge states of 2 to 8 were included.

The .RAW files were processed using MaxQuant v2.2.0.0 (15) (default parameters except as noted) with the Andromeda search engine (16), searching against K. pneumoniae subsp. *pneumoniae* K52 serotype sequences (5,126 protein sequences from UP000000265 proteome ID [2 December 2022]) from UniProt (modifications for phosphorylation: variable STY and a neutral loss of H_3_O_4_P [97.9768950 Da], with D and H amino acids; abundance was normalized to that of the total proteome). Modified and unmodified peptides were included for protein quantification using label-free quantification (LFQ) (ratio count set to 1, minimum peptide set to 2, and match between runs enabled) (17). The output files were assessed with Perseus v2.0.7.0 (18); data were filtered to remove reverse peptides, potential contaminants, and valid values (proteins present in 3 of 4 replicates under at least one condition). Phosphopeptides were filtered for localization probability of >75%, with statistical analysis with Student's *t* test (*P* value of <0.05, false discovery rate of 0.05, and *S*_0_ value of 1). For the total proteome data set, 1,895 proteins were identified (37% of the encoding proteome), and 18 phosphorylated proteins were measured (Table 1). Notably, given the acidic phosphoenrichment conditions, which limit phospho-group identification in positive-ion mode on the mass spectrometer, phosphohistidine and phosphoaspartate are underrepresented in the data set (19–21). Therefore, the extended analysis from the total proteome data set for DH modifications identified an additional five phosphopeptides.

**TABLE 1:** Phosphorylated proteins detected in K. pneumoniae under iron-limited versus iron-replete conditions

Protein identification no.	Gene name	Multiplicity	Amino acid	Position(s)	Function
STY modifications					
A6T5V4	KPN_00525	1	T	3	Putative aldo/keto-reductase
A6T671	KPN_00642	1	S	144	Biotin sulfoxide reductase
A6T6D3	*pgm*	1	S	146	Phosphoglucomutase
A6T7K6	*icdA*	1	S	113	Isocitrate dehydrogenase
A6T818	KPN_01306	2	T	331, 335	Putative transport protein
A6TAE1	*lpp*	1	S	53	Major outer membrane lipoprotein
A6TAG7	*ppsA*	1	T	419	Phosphoenolpyruvate synthase
A6TAQ3	KPN_02248	2	T	48	Type VI secretion system lipoprotein
A6TD07	KPN_03077	1	T	25	Repressor of *galETK* operon
A6TEP1	*yhcB*	1	Y	38	DUF1043 family protein
A6TEW5	*rplP*	1	T	7	50S ribosomal protein L16
A6TEX3	*rpsJ*	1	T	44	30S ribosomal protein S10
A6TG34	*glmU*	2	T	29	Bifunctional protein GlmU
DH modifications					
A6T726	*asnS*	1	D	404	Asparagine-tRNA ligase
A6T742	*rlmL*	1	D	640	rRNA large subunit methyltransferase K/L
A6TAV6	KPN_02301	1	H	264	Alpha-E domain-containing protein
A6TG01	*gyrB*	1	D	17	DNA gyrase subunit B
A6TH76	*yjeP*	1	D	406	Putative periplasmic binding protein

### Data availability.

The .RAW and affiliated files are publicly available through the PRIDE partner database for the ProteomeXchange consortium (PRIDE accession number PXD040658).
